# On the Heuristic Procedure to Determine Processing Parameters in Additive Manufacturing Based on Materials Extrusion

**DOI:** 10.3390/polym12123009

**Published:** 2020-12-16

**Authors:** Georgijs Bakradze, Egīls Arājs, Sergejs Gaidukovs, Vijay Kumar Thakur

**Affiliations:** 1 Institute of Solid State Physics, University of Latvia, LV-1063 Riga, Latvia; georgijs.bakradze@cfi.lu.lv; 2 FabControl Ltd., LV-1007 Riga, Latvia; egils@fabcontrol.com; 3 Institute of Polymer Materials Faculty of Materials Science and Applied Chemistry, Riga Technical University, LV-1048 Riga, Latvia; sergejs.gaidukovs@rtu.lv; 4 Biorefining and Advanced Materials Research Center, Scotland’s Rural College (SRUC), Kings Buildings, Edinburgh EH9 3JG, UK

**Keywords:** 3D printing, FDM, materials extrusion, process parameters, optimization, design of experiment

## Abstract

We present a heuristic procedure for determining key processing parameters (PPs) in materials-extrusion-based additive manufacturing processes. The concept relies on a design-of-experiment approach and consists of eleven “test objects” to determine the optimal combinations of key PPs values, starting with the PPs for printing the first layer and progressing to more complex geometric features, e.g., “bridges”. In each of the test objects, several combinations of the known PPs’ values are used, and only the values resulting in the best printed-part quality are selected for the following tests. The concept is intrinsically insensitive to different artefacts of the additive manufacturing machine (e.g., discrepancies between the nominal and actual nozzle diameters, and improper calibration of the feeding screws) and the optimal values of key PPs for manufacturing defect-free parts under the actual processing conditions can be determined. We validated the proposed procedure for two common commercial polymer feedstock materials, and we show that, by using the proposed procedure, it is possible to reduce the optimization time down to several hours, as well as to reduce the amount of consumed feedstock material. Tensile tests revealed a strong effect of amorphous and semi-crystalline nature of the polymer on the results of optimization. To the best of our knowledge, this is the first attempt to describe a systematic approach for optimizing PPs for materials extrusion-based additive manufacturing processes without relying on statistical data analysis or virtual simulations. The concept was implemented as a web-tool 3DOptimizer^®^.

## 1. Introduction

Additive manufacturing (AM) is no longer used to produce only prototypes, but it is slowly being established as a serial production method in the automotive, aerospace, medical, and sports industries [1,2]. AM offers major advantages over traditional manufacturing, e.g., increased part complexity and new degrees of freedom, weight reduction and materials savings, reduced assembly and cycle times, and (mass) customization opportunities [3]. Recently, many 3D printers have begun to operate using non-proprietary feedstock materials, especially in material-extrusion-based AM (MEAM), also known as fused filament fabrication (FFF) or fused deposition modeling (FDM) [2,4,5,6,7]. Along with an opportunity to design by using a wider range of engineering-grade materials, this brings new requirements to quickly and systematically determine valid process parameters (PPs).

**Figure 1:**
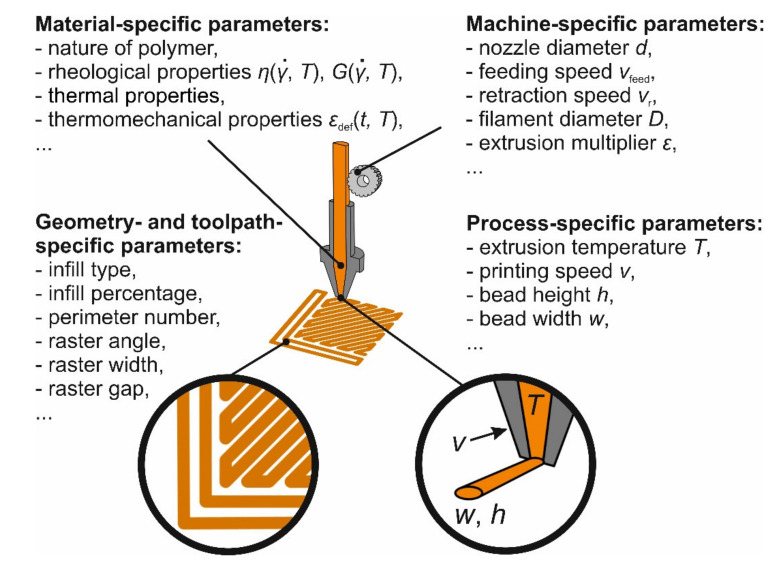
A diagram to illustrate different parameters in a material-extrusion-based additive manufacturing process.

The quality and performance of MEAM parts strongly depend on a careful selection of PPs values [2,5,8,9,10,11] (see Figure 1). The key PPs in MEAM are (i) process-specific parameters (such as printing speed, bead height, bead width, extrusion temperature, etc.), (ii) machine-specific parameters (nozzle diameter, filament diameter, feeding speed, retraction speed, extrusion multiplier, etc.), (iii) material-specific parameters (rheological properties, thermal properties, (thermo-)mechanical properties, nature of the polymer, etc.), and (iv) geometry- and toolpath-specific parameters (number of perimeters, raster angle, infill percentage, infill type, support structures, etc.). Clearly, some of these quantities are often interdependent, and the above classification cannot be considered ultimate. In the following, we discuss only key PPs from the first and second group, i.e., consider a real-life situation when one needs to manufacture a certain part with a given machine and given feedstock material.

The main aim of PPs optimization is to improve the physical and mechanical behavior of MEAM parts [2,6,7,8,12,13,14,15,16] or to reduce their processing or post-processing time [5]. In the cited papers, some PPs were varied and some performance metric of MEAM parts was measured, and several approaches (e.g., Taguchi method [13], Grey relational analysis [15], teaching-learning-based optimization algorithm [16], fuzzy logic [7], artificial neural network [17], fractional and/or full factorial analysis, etc.) have been explored, to optimize the mechanical properties of MEAM parts. However, a large amount of consistent data is required to implement the described optimization approaches, which limits their use in real-life applications of MEAM. Added to that, the lack of general standards complicates meaningful comparisons between AM machines, feedstock materials, and final properties of MEAM parts. This results in a situation in which AM engineers have to arrive at the optimal PPs’ values intuitively rather than purposefully. Therefore, an urge exists to develop a consistent practical procedure to find the optimal PPs’ values.

This paper presents and experimentally validates a practical method that allows finding the PPs’ values for processing feedstock materials, using open-materials MEAM printers. A design-of-experiment (DOE) based optimization approach was implemented in a web-based software tool, 3DOptimizer^®^, that generates adaptive toolpath files—.GCODE files—with test geometries, to 3D print by using a given combination of an AM machine and feedstock material. By visual inspection of the 3D-printed structures, one can identify the optimal PPs’ values. To the best of our knowledge, this is the very first attempt to describe a simple systematic approach for optimizing key PPs in MEAM processes, without relying on statistical models or computationally costly simulations.

## 2. Materials and Methods

A MEAM 3D printer (XD 20 by Mass Portal, Riga, Latvia) with a cylinder build volume (height × diameter: 250 mm × 200 mm) and an enclosed build chamber was used. The 3D printer is equipped with precision miniature linear guideways, and the stepper motors allow an XYZ accuracy of 15 µm, 15 µm, 5 µm, respectively. The printing head has 3 fans to cool down deposited layers. The temperature of the nozzle and build plate is controlled by a type-K thermocouple and reported as is (i.e., without any re-calibration factors). We used a brass nozzle with an inner diameter of 0.8 mm, a capillary length of 5 mm, and a cone angle of 60°. Polyamide (PA) and acrylonitrile butadiene styrene (ABS) based natural color 3D printing filaments with the outer diameter of 1.75 mm were used: PA6/66-based Novamid^®^ ID 1030 (Nexeo Solutions 3D, Barcelona, Spain) and ABS-based CYCOLACTM AMMG94F (Nexeo Solutions 3D, Barcelona, Spain). In the following, these materials are designated as PA and ABS, respectively. Before printing, filaments were dried to constant weight for 7 h at 75 °C. The melt flow indexes are 2.6 g/10 min (2.16 kg) and 11.7 g/10 min (3.8 kg), as determined at 230 °C (ASTM D1238) for PA and ABS, respectively. The melting point of PA is 200 °C (as determined by DSC at 10 °C/min (STAR system DSC-1 by Mettler Toledo, Columbus, OH, USA)).

The .STL models of the 1B-type standard specimens were processed by using Slic3r Prusa Edition 1.33.3 [18] to generate toolpaths files. The specimens were printed without supports, with no top or bottom solid layers, using the maximum number of perimeters (i.e., the 3D-printed specimens effectively had a nominal density of 100%). As no mechanical testing standards exist for MEAM parts, ISO 527 guidelines were referenced for mechanical testing. At room temperature, the tensile tests were performed on a universal testing machine (25ST by Tinius Olsem, Horsham, PA, USA) equipped with a load cell. Four to five specimens were tested for each printing condition, at the crosshead speed of 2 mm/min. The specimens were tested in an as-printed condition, at least 48 h after printing. The modulus of elasticity, tensile strength, and the tensile strain were calculated from the recorded stress–strain curves.

## 3. General Description of the Testing Procedure

### 3.1. Test Objects

The practical method encompasses eleven print tests whose sequence is justified in Supplementary Materials Section 1; some of the tests are optional (see Table 1 for details).

**Figure 2:**
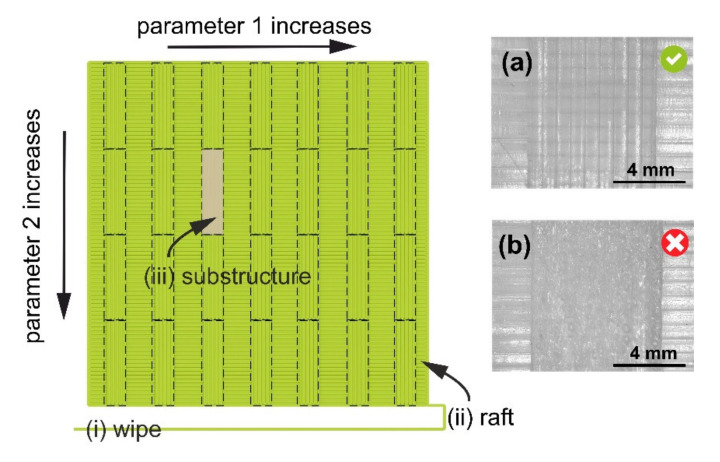
Test geometry consists of (top view) (i) a wipe strip, (ii) a square raft structure to support (test structures), and (iii) 28 test structures. The values of Parameter 1 increase in columns, from right to left; the values of Parameter 2 increase in rows, from top to bottom. Insets show micrographs of two substructures in an extrusion temperature vs. printing speed test for PA: (**a**) and (**b**) correspond to optimal (268 °C and 25 mm/s) and non-optimal (290 °C and 15 mm/s) PP values, respectively. Other PP values: bead height, 0.20 mm; and nominal bead width, 0.80 mm.

Figure 2 shows a computer-rendered view of a test geometry which was used in several tests: extrusion temperature vs. printing speed (#3 in Table 1), bead height vs. printing speed (#4), bead width vs. printing speed (#5), and extrusion multiplier vs. printing speed test (#6). The test object consists of the following: (i) a wiping strip to prime the nozzle, (ii) a solid support layer (“raft” for the test substructures), and (iii) test substructures. The bottom solid layer is not used in the tests to optimize PPs for printing the first layer, e.g., first-layer bead height and first-layer bead width tests (#1 and #2 in Table 1, respectively). In first-layer bead height and first-layer bead width tests, the test substructures consist of a single layer and are printed on the print bed; in all other tests, the test substructures consist of four layers and are supported on a bottom solid layer. Typically, in a single print test, 28 combinations of two PPs are tested: In the columns, Parameter 1 accepts one out of seven evenly distributed values from right to left, while in the rows, Parameter 2 accepts one out of four evenly distributed values from top to bottom (see the main body of Figure 2). The default value ranges for different PPs can be found in Table 2; these values were determined empirically, based on a vast 3D-printing experience of the authors. Different PPs do not have to be optimized concurrently, for their mutual dependence is separated in each test (e.g., bridging settings are irrelevant for printing the first layer). The PP values selected in a current test are used to generate a .GCODE file for the next one.

**Figure 3:**
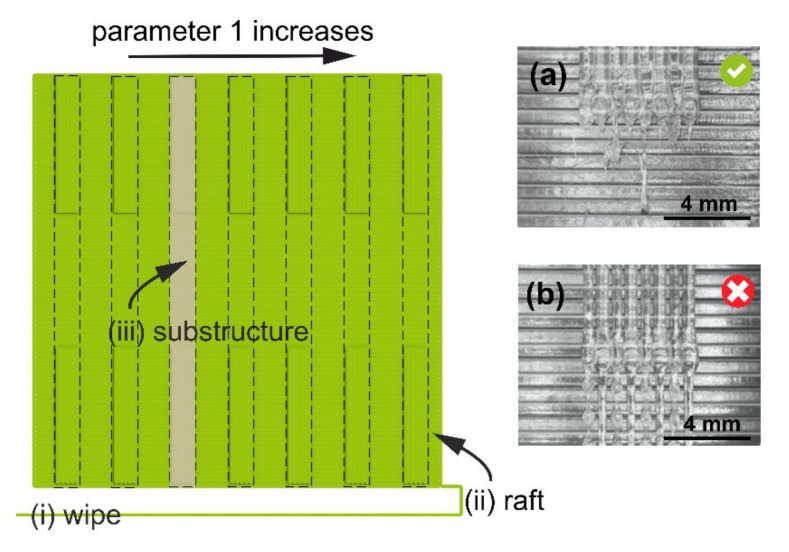
Test geometry for the retraction distance test (#10) consists of (top view) (i) a wipe strip, (ii) a square raft structure to support (substructures), and (iii) seven test substructures. The values of the retraction distance (Parameter 1) increase from left to right. The insets show micrographs of two substructures in a test for PA: (**a**) and (**b**) correspond to an optimal (4.00 mm) and not optimal (1.00 mm) value of the retraction distance, respectively. Other PP values: bead height, 0.20 mm; extrusion temperature, 268 °C; printing speed, 25 mm/s; retraction speed, 120 mm/s; nominal bead width, 0.80 mm.

**Figure 4:**
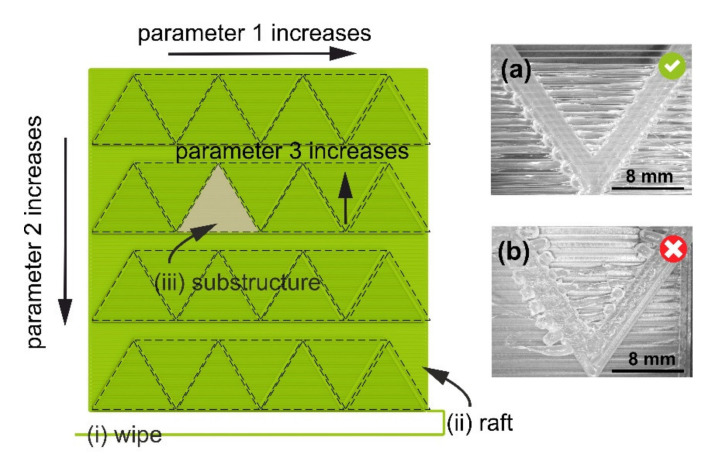
Test geometry for the bridging test (#11) consists of (top view) (i) a wipe strip, (ii) a square raft structure with zigzag walls (supporting substructures), and (iii) bridging test substructures of varying length. The values of the bridging extrusion multiplier (Parameter 1) increase from left to right, the bridging printing speed (Parameter 2) increases from top to bottom, and the bridging length (Parameter 3) increases within the substructure. Insets show micrographs of two substructures in a test for PA: (**a**) and (**b**) correspond to optimal (bridging extrusion multiplier of 2.0 and bridging printing speed of 35 mm/s) and non-optimal (bridging extrusion multiplier of 1.0, bridging printing speed of 25 mm/s) PP values, respectively. Other PP values: bead height, 0.20 mm; extrusion temperature, 268 °C; printing speed, 25 mm/s; nominal bead width, 0.80 mm.

In the retraction test (#8) and bridging test (#11), a possibility exists to test three PPs at the same time: In this case, the value of the third PP (retraction speed or bridging length, respectively) is increasing within the substructure (i.e., each bead is printed by using a different value of Parameter 3). The main bodies of Figure 3 and Figure 4 show the test geometries used in retraction (#10) and bridging tests (#11), respectively. For optimal performance in all tests, the length of the substructure should be comparable to the characteristic size of the actual object or part to be printed. A .GCODE file with the geometry for each test is line-by-line assembled by a custom-written Python script.

### 3.2. Criteria for Selecting Optimal PP Values

**Figure 5:**
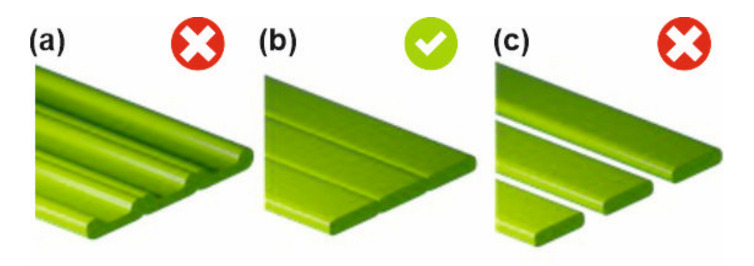
Beads building up a solid layer: (**a**) the extruded amount is too high (over-extrusion); (**b**) the bead height, the printing speed, and the extruded amount are well-balanced; (**c**) the extruded amount is too low (under-extrusion) to create a closed layer.

When a generated .GCODE file has been printed, the print has to be inspected, and the optimal values of PPs have to be selected. Some combinations of PP values will result in a better quality of test substructures; this is the case when individual beads create a smooth solid layer with no gaps in between (see Figure 5c). If the extruded amount is too high, the beads will make grooves (Figure 5a); when the extruded amount is too low (under-extrusion), a layer will have gaps (Figure 5c).

The beads should have uniform heights and widths, with good melt-together, and no bubbles or color changes. In retraction tests (#8, #9, and #10), an additional criterion is the minimum amount of “strings” (cf. Figure 3a,b). In Figure 3b, the retraction distance is not optimal, and the nozzle is losing material in the form of “strings”. In the bridging test (#11), an additional criterion is that the bridges (i.e., unsupported structures) should be continuous and flat (cf. Figure 4a,b). In Figure 4b, the combination of bridging speed and bridging extrusion multiplier values is not optimal, and continuous bridges fail to form. In each test, the methodology allows for identifying optimal PP values which result in the best-looking test substructures under the actual processing conditions (feedstock material, machine, and environment). Thus, if one uses the validated PP values, serious build defects (voids and under- or over-extrusion) will be naturally eliminated in the parts manufactured by MEAM.

### 3.3. Optimization Strategies

One can identify three different requirements for a MEAM part or MEAM process: (i) low surface roughness, (ii) good mechanical properties (at the expense of geometrical precision and good cosmetic properties), or (iii) short printing time (at the expense of surface roughness and mechanical strength). We refer to these requirements as optimization strategies for aesthetics, mechanical strength, and short printing time, respectively. The rationale behind the optimization procedure is based on the following qualitative considerations: Printing at lower speeds and (reasonably) high temperatures results in a better polymer chain diffusion and a better bond strength and, thus, favors mechanical strength. Printing at lower temperatures, with lower bead heights and lower printing speeds, favors optimization for aesthetics. Printing at higher temperatures, with higher printing speeds, results in a short printing time but will also lead to a distorted or less precise geometry. Therefore, depending on the optimization strategy, one has to evaluate the quality of substructures by using slightly different criteria. For example, when having several substructures of equally good quality, systematic validation of the results with higher printing speed would result in shorter printing times, whereas a systematic validation of the results with lower extrusion temperature and lower printing speed would result in enhanced surface quality.

## 4. Results and Discussion

**Figure 6:**
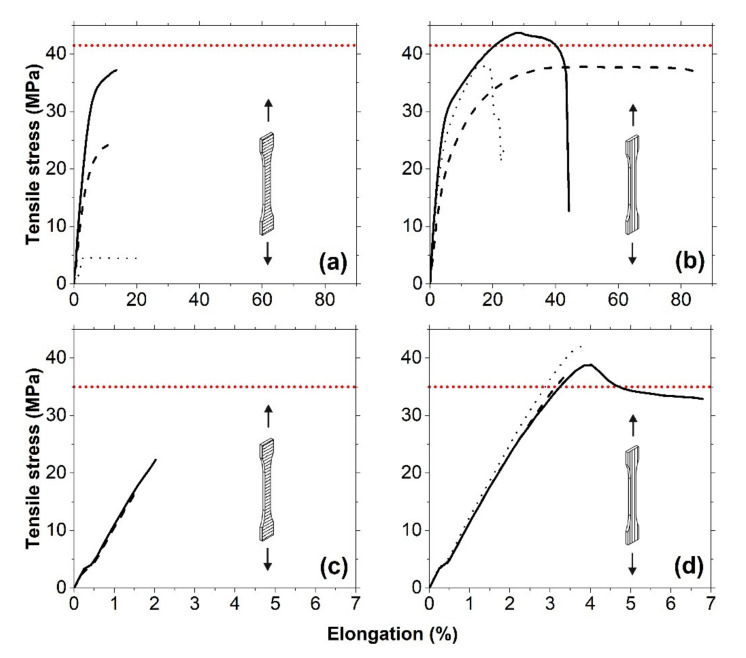
Typical stress–strain curves for tensile bars manufactured from PA (**a**,**b**) and ABS (**c**,**d**), using Table S2 in Supplementary Materials. The loading direction is normal (**a**,**c**) and parallel (**b**,**d**) to the weld interfaces. The solid, dashed, and dotted lines correspond to optimization strategies for mechanical strength, short printing time, and aesthetics, respectively. The red horizontal dotted line shows the ultimate tensile strength of monolith specimens obtained by injection moulding.

To validate the outlined procedure, we used the optimized PPs values (see Table S2 in Supplementary Materials Section 2) as input values for the slicing software, to process the .STL models of the standard tensile bars, i.e., to generate the toolpath files, which were later used to manufacture the tensile bars by MEAM. Table 3 summarizes the results of tension tests of standard tensile bars from PA (a) and ABS (b) with two different buildup directions and loading schemes: transversal (when the loading direction is parallel to the buildup direction, i.e., is perpendicular to the weld interfaces) and longitudinal (when the loading direction is perpendicular to the buildup direction, i.e., parallel to the weld interfaces). As it is apparent from Table 3, the dimensions of the tensile bars produced by MEAM deviated from the nominal dimensions, and the best correspondence was observed for the parts printed by using the PP values optimized for aesthetics. Despite the geometrical similarity, their mechanical properties varied depending on the optimization strategy. The following can be seen from Table 3: (i) The PP values optimized for mechanical strength, indeed, result in the stronger parts; (ii) the PP values optimized for a short printing time require the minimum time per part to be printed, yet result in the less strong parts; and (iii) the PP values optimized for aesthetics result in the weakest parts, but with a narrower spread in the dimensions. Let us discuss the typical tensile stress–strain curves for the PA specimens in more detail (see Figure 6a,b).

The ultimate tensile stress of the PA specimens under the load applied normally to the weld interfaces is very sensitive to the selected optimization strategy: It can be as low as 4.8 MPa (this result should not be considered as a failed print, because the part has been fully manufactured without visible printing defects) when processed by using the PP values optimized for aesthetics, and it can reach its maximum value of 34.5 MPa when processed by using the PP values optimized for mechanical strength (see Figure 6a). Although the PA specimens processed by using the PP values optimized for aesthetics show the worst mechanical properties, they have a good surface finish. Inter-layer cracking and delamination in these specimens under the applied load hint to residual stresses formed due to a large number of heating/cooling cycles.

When tensile stress is applied normally to the weld interface, the PA specimens show a combination of elastic and plastic deformation, with strain values in the range of 10–20%. The ultimate tensile stress of the specimens printed under the load applied parallel to the weld interfaces is not that sensitive to the selected optimization strategy, and, in all cases, it is between 37.5 and 42.4 MPa (see Figure 6b). When tensile stress is applied parallel to the weld interface, the deformation mechanism of PA specimens strongly depends on the chosen optimization strategy. As it is apparent from Figure 6b, optimizing PPs for a short printing time results in highly ductile specimens with the permanent plastic deformation and tensile strain of about 80%. If optimized for mechanical strength, the strain values decrease by a factor of two, while their stiffness and tensile strength increase. The specimens optimized for aesthetics reveal the lowest tensile deformation values.

Typical tensile stress–strain curves for the ABS specimens are shown in Figure 6c,d. The stress–strain curves for ABS specimens under the load applied normally to the weld interfaces do not differ and are independent of the chosen printing optimization strategy (see Figure 6c). The ultimate stress of the specimens is also not very sensitive to the selected optimization strategy but considerably depends on the loading direction: 19.6 and 37.6 MPa for the specimens loaded normally and parallel to the weld interface, respectively (see Table 3). No macroscopic interlayer cracking or delamination was observed in the specimens; the fracture occurred in the mixed (adhesive/cohesive) mode. The failure mode for all specimens under the load applied normally to the weld interfaces was brittle without any plastic deformation region. If optimized for the short printing time or aesthetics, the ABS specimens under the load applied parallel to the weld interfaces show brittle behavior; however, if optimized for mechanical strength, the specimens plastically deform with a clear phenomenon of yielding and an apparent decrease in stress, followed by material deformation around some constant stress value until the failure. The tensile strain values are in the range of 3–4%.

The mechanical properties of the parts made of semi-crystalline PA depend on the PP values more than those of the parts made of amorphous ABS. For both polymers, the PP values optimized for mechanical strength, indeed, result in stronger parts. As it was shown in Reference [19], voids and their shape have a crucial influence on the local stress distribution and, thus, mechanical properties of the MEAM processed parts. As the criteria for selecting PPs’ values in each optimization step rely on the visual inspection (e.g., absence of the voids and uniformity), the parts manufactured by using the optimized PP values have better structural integrity and, thus, better mechanical properties. As the MEAM is inherently a highly non-stationary process, the microstructure of the printed parts can be very complex [20], the simple qualitative approach described above clearly cannot be considered as the ultimate optimization procedure for MEAM; however, it has a benefit that it quickly provides printing settings which purposely lead to desired properties of the MEAM part (aesthetics, mechanical strength) or MEAM process (short printing time). The end-users of “open-materials” MEAM machines can benefit from using the described practical method in several ways: (i) when determining PP values for processing a new feedstock material using available MEAM machines, (ii) when determining PP values for processing available feedstock materials using a new MEAM machine, and (iii) when adjusting the already determined PP values to different manufacturing conditions. The optimized PP values can also be used as input values for finite-element-analysis-based models. The procedure can be further refined to make the selection of the optimal values of key PPs more objective (e.g., by measuring the profiles of the grooves with a profilometer or implementing an image recognition system, etc.) or to include geometry- and toolpath-specific parameters [21]. The procedure itself can become a useful add-on feature in a toolpath-generation software or 3D printers. Similar optimization procedures have been put forward to optimize printing parameters in photo-polymerization and powder-bed-fusion processes.

## 5. Conclusions

A heuristic, practical method for finding optimal values of key PPs in MEAM processes was developed. The main idea of the proposed method is to generate custom toolpath files in which several known PP values are being used sequentially and the PP values resulting in the best quality are selected upon printing generated toolpath files. This DOE-based methodology allows us to promptly determine optimal values of key PPs for manufacturing defect-free parts under the actual processing conditions. Because the mutual influence of PPs is separated in individual tests, and because up to 28 different value combinations of PPs can be tested in a single print, the whole optimization time could be greatly reduced and varied between 127 and 200 min, depending on the optimization strategy. In each optimization procedure, less than 20 m of 3D-printing filament was consumed. The results demonstrated that it is possible to purposefully optimize the PP values in MEAM processes with respect to cosmetic properties, printing time, or mechanical properties of the 3D-printed parts, without compromising the stability of the MEAM process.

## Figures and Tables

**Table 1:** The sequence of tests to optimize key processing parameters (PPs) in material-extrusion-based additive manufacturing MEAM. List of pairs or triples of key PPs and geometry type used in each test. The optional tests are marked with an asterisk (*).

Number	Parameter 1	Parameter 2	Parameter 3	Geometry
1	First-layer bead height	First-layer printing speed	–	1
2 *	First-layer bead width	First-layer printing speed	–	1
3	Extrusion temperature	Printing speed	–	1
4 *	Bead height	Printing speed	–	1
5 *	Bead width	Printing speed	–	1
6 *	Extrusion multiplier	Printing speed	–	1
7 *	Printing speed	–	–	1
8	Extrusion temperature	Retraction distance	Retraction speed	2
9 *	Retraction distance	Printing speed	–	2
10 *	Retraction distance	–	–	2
11	Bridging extrusion multiplier	Bridging printing speed	Bridging distance	3

**Table 2:** Default value ranges for key PPs in MEAM for common polymers.

The Parameter Name (Units)	Value Range	Note
First-layer bead height (mm)	(0.30*d*, 0.70*d)* ^1^ (0.25*d*, 0.50*d)*	*d* > 0.4 mm *d* ≤ 0.4 mm
First-layer printing speed (mm/s)	(10, 30) (5, 15)	for Bowden extruders for direct drive extruders
First-layer bead width (mm)	(0.90*d*, 1.25*d)*	-
Extrusion temperature (°C)	*(T*_1_, *T*_max)_^2,3^	-
Bead height (mm)	(0.50*d*, 0.75*d)*	can be defined by user
Bead width (mm)	(0.82*d*, 1.45*d)*	-
Extrusion multiplier (-)	(0.85, 1.15) (0.95, 1.25)	for hard polymers for soft polymers
Printing speed (mm/s)	(25, 55) (15, 40)	for Bowden extruders for direct drive extruders
Retraction distance (mm)	(0.0, 6.0) (0.0, 3.0)	for Bowden extruders for direct drive extruders
Retraction speed (mm/s)	(60, 120) (30, 80)	for Bowden extruders for direct drive extruders
Bridging extrusion multiplier (-)	[1.00, 2.00)	-
Bridging printing speed (mm/s)	(0.25*v*, 0.75*v)* ^4^	-

^1^*d*—nozzle inner diameter, ^2^*T*_1_—extrusion temperature of the first layer, ^3^*T*_max_—maximum extrusion temperature, ^4^*v*—printing speed value selected in previous tests.

**Table 3:** Results of tension tests of standard tensile bars 3D-printed with PA (a,b) and ABS (c,d), using the optimized PP values. The loading direction is parallel (a,c) and normal (b,d) to the buildup direction. The number of layers and the average printing time per single part are shown too. The mechanical properties of the injection-molded parts are listed in the column “Monolith” (as reported in the manufacturer-supplied technical documentation sheets).

(a) PA: parallel to buildup direction (normal to weld interfaces)				
	**Aesthetics**	**Short Printing Time**	**Mechanical Strength**	**Monolith**
Width (mm)	9.80 ± 0.02	10.15 ± 0.06	10.01 ± 0.07	10.00
Thickness (mm)	4.20 ± 0.03	4.40 ± 0.08	4.14 ± 0.08	4.00
Tensile modulus (MPa)	98.9 ± 53	445 ± 115	806 ± 132	2330
Ultimate strain (%)	3.3 ± 0.8	16.0 ± 6.2	8.2 ± 3.1	>50
Ultimate stress (MPa)	4.8 ± 1.0	27.4 ± 2.3	34.5 ± 2.0	41.5
Printing time per part (min)	63	17	39	–
Number of layers (-)	1498	600	749	–
(b) PA: normal to buildup direction (parallel to weld interfaces)				
	**Aesthetics**	**Short Printing Time**	**Mechanical strength**	
Width (mm)	10.20 ± 0.02	10.25 ± 0.05	10.30 ± 0.07	
Thickness (mm)	4.03 ± 0.03	4.08 ± 0.06	3.87 ± 0.05	
Tensile modulus (MPa)	785 ± 53	623 ± 53	824 ± 77	
Ultimate strain (%)	17.4 ± 1.0	46.6 ± 8.8	27.7 ± 0.5	
Ultimate stress (MPa)	40.1 ± 0.8	37.5 ± 0.7	42.4 ± 1.1	
Printing time per part (min)	57	21	27	
Number of layers (-)	38	16	19	
(c) ABS: parallel to buildup direction (normal to weld interfaces)				
	**Aesthetics**	**Short Printing Time**	**Mechanical Strength**	**Monolith**
Width (mm)	10.15 ± 0.10	10.28 ± 0.05	10.28 ± 0.05	10.00
Thickness (mm)	4.15 ± 0.05	4.23 ± 0.03	4.29 ± 0.05	4.00
Tensile modulus (MPa)	1290 ± 81	1255 ± 57	1307 ± 51	2450
Ultimate strain (%)	1.6 ± 0.1	2.0 ± 0.2	1.8 ± 0.3	18
Ultimate stress (MPa)	17.7 ± 1.3	21.8 ± 1.8	19.3 ± 2.9	35
Printing time per part (min)	37	25	37	–
Number of layers (-)	749	749	749	–
(d) ABS: normal to buildup direction (parallel to weld interfaces)				
	**Aesthetics**	**Short Printing Time**	**Mechanical Strength**	
Width (mm)	10.33 ± 0.05	10.43 ± 0.05	10.58 ± 0.19	
Thickness (mm)	4.01 ± 0.06	4.00 ± 0.00	4.35 ± 0.09	
Tensile modulus (MPa)	1483 ± 88	1340 ± 83	1540 ± 36	
Ultimate strain (%)	3.5 ± 0.4	3.4 ± 0.4	3.3 ± 0.3	
Ultimate stress (MPa)	38.4 ± 2.1	35.5 ± 2.5	39.0 ± 2.3	
Printing time per part (min)	43	31	35	
Number of layers (-)	19	19	19	

## Supplementary Materials

The following file is available online at https://www.mdpi.com/2073-4360/12/12/3009/s1. Table S1: Typical values of selected PP in MEAM and their resulting influence on the change in the volumetric ow rate. Table S2: Key PPs optimized for MEAM processing of PA (a) and ABS (b) using Mass Portal XD20 3D printer with an 0.8-mm nozzle. The length of the consumed lament and the total printing time is shown as well. The values which were determined in a test are marked with an asterisk.
