# Supplementary material for: On the Heuristic Procedure to Determine Processing Parameters in Additive Manufacturing Based on Materials Extrusion
Source: Polymers (Basel). 2020 Dec 16;12(12):3009. doi: 10.3390/polym12123009 (PMC7766196; doi:10.3390/polym12123009)
Supplement: Supplementary file 1 [file polymers-12-03009-s001.pdf]

# Supplementary information for: On the heuristic procedure to determine processing parameters in additive manufacturing based on material extrusion

October 21, 2020

## 1 Test sequence and its justification

The equation for the nominal volumetric flow rate (in $mm^3/s$) reads as follows (Jin et al. 2015): $q = \epsilon v\Big[(w-d)h + \frac{\pi}{4}h^2\Big]$ , where $\epsilon$ is the extrusion multiplier, $v$ is the printing speed, $w$ is the bead width, $h$ is the height, and $d$ is the nozzle inner diameter. Let us estimate how sensitive the volumetric flow rate $q$ is with respect to the change of a PP (i.e., extrusion temperature $T$, $v$, $\epsilon$, $w$ or $h$). For that we use the definition of a derivative and from the expression for the flow rate it follows immediately for $\Delta q$:

$$\Delta q_\epsilon = \frac{\partial q}{\partial \epsilon}\Delta\epsilon = v\Big[(w-d)h + \frac{\pi}{4}h^2\Big]\Delta\epsilon \approx \frac{\pi}{4}h^2 v\Delta\epsilon,$$

$$\Delta q_v = \frac{\partial q}{\partial v}\Delta v = \epsilon\Big[(w-d)h + \frac{\pi}{4}h^2\Big]\Delta v \approx \frac{\pi}{4}h^2 \epsilon\Delta v,$$

$$\Delta q_w = \frac{\partial q}{\partial w}\Delta w = \epsilon v h\Delta w,$$

$$\Delta q_h = \frac{\partial q}{\partial h}\Delta h = \epsilon v\Big[(w-d) + \frac{\pi}{2}h\Big]\Delta h \approx \frac{\pi}{2}h\epsilon v\Delta h.$$

It is less straightforward to estimate the influence of the extrusion temperature on the volumetric flow rate. However, by adopting the approach developed by (Mackay et al. 2017) and taking into account the values for PA used in this paper and the typical change steps (see Table 1) one gets: $\Delta q_\epsilon < \Delta q_v < \Delta q_w < \Delta q_h < \Delta q_T$; this gives a rough estimate about the influence of these PP on the flow rate and also explains the test sequence: PPs are optimized in accordance to their influence on the flow rate.

**Place for Table 1.**

Moreover, the proposed test sequence allows to separate the mutual influence of the PPs (e.g., bridging printing settings are irrelevant for printing the first layer and vice versa, etc.). From all the PPs discussed above, a change in the

extrusion temperature has the most pronounced influence on the change in the flow rate. Obviously for a given material and hotend/nozzle combination, there is a maximum flow rate which corresponds to the maximum printing speed.

# 2 Optimization results for PA and ABS

For both 3D printing materials we have performed three sets of optimization routines aiming for aesthetics, short printing time, and mechanical strength, respectively. It normally took about 3 hours and less than 20 m of the filament to perform an optimization routine (see Table 2). The total printing time in case of PA optimization for aesthetics is longer because an optional bead width test had to be performed. This had little influence on the total consumed filament length: a bead height value of 0.10 mm was used when optimizing for aesthetics, whereas the bead height values of 0.20 mm and 0.25 mm were used when optimizing for short printing time or mechanical strength, respectively. For ABS we have fixed the bead height to 0.20 mm for all three optimization scenarios. Table 2 summarizes the PP values which were determined (marked with an asterisk) or defined in a test.

**Place for Table 2.**

# References

Jin, Y., Li, H., He, Y. & Fu, J.-Z. (2015), 'Quantitative analysis of surface profile in fused deposition modelling', *Additive Manufacturing* **8**, 142 – 148.
**URL:** *http://www.sciencedirect.com/science/article/pii/S2214860415000512*

Mackay, M. E., Swain, Z. R., Banbury, C. R., Phan, D. D. & Edwards, D. A. (2017), 'The performance of the hot end in a plasticating 3D printer', *Journal of Rheology* **61**, 229 – 236.
**URL:** *https://doi.org/10.1122/1.4973852*

Table 1: Typical values of selected PP in MEAM and their resulting influence on the change in the volumetric flow rate.

| PP (units) | Typical value | Typical change step | Change in flow rate ($mm^3/s$) |
|---|---|---|---|
| Extrusion multiplier (-) | 1.00 | 0.05 | 0.04 |
| Printing speed (mm/s) | 25 | 5 | 0.16 |
| Bead width (mm) | 0.8 | 0.05 | 0.25 |
| Bead height (mm) | 0.2 | 0.05 | 1.96 |
| Extrusion temperature (℃) | 240 | 5 | 15.6 |

Table 2: Key PPs optimized for MEAM processing of PA (a) and ABS (b) using Mass Portal XD20 3D printer with an 0.8-mm nozzle. The length of the consumed filament and the total printing time is shown as well. The values which were determined in a test are marked with an asterisk.

| PP (units) | Aesthetics | Short printing time | Mechanical strength |
|---|---|---|---|
| *(a) PA* | | | |
| Build plate temperature (°C) | 100 | 100 | 100 |
| First layer extrusion temperature (°C) | 240 | 240 | 240 |
| First layer printing speed (mm/s) | 16.7* | 16.7* | 16.7* |
| First layer bead height (mm) | 0.33* | 0.33* | 0.33* |
| First layer bead width (mm) | 0.80 | 0.80 | 0.80 |
| Extrusion temperature (°C) | 246* | 290* | 268* |
| Bead height (mm) | 0.10* | 0.25* | 0.20* |
| Bead width (mm) | 0.93* | 0.80 | 0.80 |
| Extrusion multiplier (–) | 1.00 | 1.00 | 1.00 |
| Printing speed (mm) | 30.0* | 50.0* | 25.0* |
| Retraction distance (mm) | 4.00* | 0.667* | 4.00* |
| Retraction speed (mm/s) | 120 | 120 | 120 |
| Cooling power (%) | 100 | 100 | 0 |
| Bridging extrusion multiplier (–) | 1.75* | 1.75* | 1.75* |
| Bridging printing speed (mm/s) | 15.0* | 25.0* | 12.0* |
| Bridging cooling power (%) | 100 | 100 | 100 |
| Filament consumed (m) | 13.2 | 14.1 | 12.9 |
| Total testing time (min) | 175 | 127 | 139 |
| *(b) ABS* | | | |
| Build plate temperature (°C) | 80 | 80 | 80 |
| First layer extrusion temperature (°C) | 250 | 250 | 250 |
| First layer printing speed (mm/s) | 15.0* | 15.0* | 15.0* |
| First layer bead height (mm) | 0.32* | 0.32* | 0.32* |
| First layer bead width (mm) | 0.80 | 0.80 | 0.80 |
| Extrusion temperature (°C) | 250* | 267* | 258* |
| Bead height (mm) | 0.20 | 0.20 | 0.20 |
| Bead width (mm) | 0.80 | 0.80 | 0.80 |
| Extrusion multiplier (–) | 1.00 | 1.00 | 1.00 |
| Printing speed (mm) | 26.7* | 48.3* | 33.3* |
| Retraction distance (mm) | 2.67* | 3.33* | 2.67* |
| Retraction speed (mm/s) | 120 | 120 | 120 |
| Cooling power (%) | 100 | 60 | 0 |
| Bridging extrusion multiplier (–) | 2.00* | 2.00* | 2.00* |
| Bridging printing speed (mm/s) | 13.0* | 24.0* | 16.0* |
| Bridging cooling power (%) | 100 | 100 | 100 |
| Filament consumed (m) | 17.6 | 12.3 | 12.3 |
| Total testing time (min) | 200 | 130 | 194 |
